# Airway responses and inflammation in subjects with asthma after four days of repeated high-single-dose allergen challenge

**DOI:** 10.1186/1465-9921-13-78

**Published:** 2012-09-19

**Authors:** Johannes Schulze, Sandra Voss, Ulrich Zissler, Markus A Rose, Stefan Zielen, Ralf Schubert

**Affiliations:** 1 Department of Allergy, Pulmonology, and Cystic Fibrosis, Children's Hospital, Goethe-University, Frankfurt, Germany

**Keywords:** Bronchial allergen challenge, Bronchial hyperresponsiveness, Exhaled NO, Eosinophils, IL-5, IL-8, IFN-γ, Foxp3

## Abstract

**Background:**

Both standard and low-dose allergen provocations are an established tool in asthma research to improve our understanding of the pathophysiological mechanism of allergic asthma. However, clinical symptoms are less likely to be induced. Therefore, we designed a protocol for repetitive high-dose bronchial allergen challenges to generate clinical symptoms and airway inflammation.

**Methods:**

A total of 27 patients aged 18 to 40 years with positive skin-prick tests and mild asthma underwent repetitive high-dose allergen challenges with household dust mites for four consecutive days. Pulmonary function and exhaled NO were measured at every visit. Induced sputum was analysed before and after the allergen challenges for cell counts, ECP, IL-5, INF-γ, IL-8, and the transcription factor Foxp3.

**Results:**

We found a significant decrease in pulmonary function, an increased use of salbutamol and the development of a late asthmatic response and bronchial hyperresponsiveness, as well as a significant induction of eNO, eosinophils, and Th-2 cytokines. Repeated provocation was feasible in the majority of patients. Two subjects had severe adverse events requiring prednisolone to cope with nocturnal asthma symptoms.

**Conclusions:**

Repeated high-dose bronchial allergen challenges resulted in severe asthma symptoms and marked Th-2-mediated allergic airway inflammation. The high-dose challenge model is suitable only in an attenuated form in diseased volunteers for proof-of-concept studies and in clinical settings to reduce the risk of severe asthma exacerbations.

**Trial registration:**

ClinicalTrials.govNCT00677209

## Background

Bronchial allergen provocation models are well established in asthma research and allow the evaluation of antiallergic and antiasthmatic agents in relatively small sample sizes [1,2]. Two different approaches to investigate the action of a drug exist. The classical approach is when subjects are challenged with an allergen before and after treatment with antiallergic or antiasthmatic drugs [3-6] and they are selected to develop a reproducible early and late asthmatic response (EAR and LAR) as well as changes in sputum parameters. Usually subjects do not develop subsequent asthma symptoms or an increased medication use.

The other provocation model imitates the natural allergen exposure. On consecutive days, a small amount of an allergen is inhaled to induce bronchial inflammation. In previous studies that used this approach, the subjects developed increased exhaled nitric oxide (eNO) and increases in the levels of eosinophils and Th-2 derived cytokines [7-14]. However, repeated low-dose allergen challenges did not provoke significant asthma symptoms with changes in FEV_1_ and induction of an LAR.

In a high-allergen provocation model provided by Grainge and Howarth [15], three consecutive challenges were performed at 48-h intervals. We hypothesised that four consecutive challenges in one week may be more likely to induce symptoms and an allergen-driven asthma exacerbation in diseased volunteers. Such a challenge model not only allows for the investigation of the airway responses and inflammation, but it also provides a strong indication of whether the clinical symptoms/exacerbations can be reduced in proof-of-concept studies in small patient samples. Therefore, repeated high-dose allergen challenges might combine both models, specifically, the induction of asthma with obstruction of the airways and significant changes in the forced expiratory volume in 1 second (FEV_1_) as well as the induction of airway inflammation with changes of eNO, eosinophils, and cytokines in sputum.

In repeated challenges, safety concerns support an incremental approach in which the allergen response can be monitored between increasing doses [16]. However, the single-step method may provide certain distinct advantages, e.g., the ability to allow for the exact and equivalent timing of allergen administration between subjects. Moreover, in studies of the inflammatory response, the single-step challenge may ensure that a constant allergen dose is delivered at any given time [2].

New antiallergic drugs are emerging, and the high-dose provocation model might be a tool to detect the potency of the agent in a short period and in a small sample size. We investigated volunteers who were allergic to household dust mites using repeated high-dose allergen provocations. In the provocation period, the use of salbutamol, changes in FEV_1_, eNO, bronchial hyperresponsiveness (BHR) and inflammatory cytokines in induced sputum were investigated.

## Methods

### Subjects and selection

The present study was part of the study “Safety, tolerability, and impact on allergic inflammation of autologous E.coli autovaccine in the treatment of house dust mite asthma - a prospective open clinical trial” [17]. Participants were screened to have a positive skin-prick test and a positive bronchial challenge against household dust mites, as well as increases in eNO and in sputum cells and cytokines. Twenty-seven subjects were invited to undergo repeated high-dose allergen challenges. All had mild asthma (GINA 1°) and subjects who used a regular therapy with inhaled or oral corticosteroids, long-acting beta-agonists, or leukotriene receptor antagonists were excluded.

All participants supplied written informed consent prior to the study. Human experimentation guidelines of Good Clinical Practice, the German Drug Act and the declaration of Helsinki/Hong Kong were followed in the conduct of clinical research.

The study had been approved by the ethical committee of the University of Frankfurt.

ClinicalTrials.govNCT00677209

### Study design

**Figure 1:**
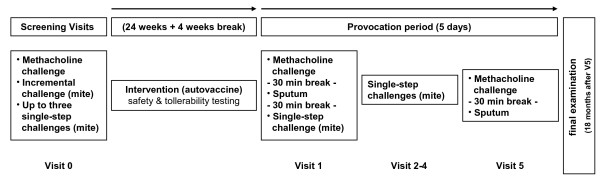
Study design.

Our cross-sectional study encompassed screening visits and a challenge period with five consecutive visits (visits 1 – 5), eight months apart (Figure 1.). At the screening visits, an initial bronchial challenge with incremental doses of mite allergen was performed to define the dose of allergen that caused a 15% drop in FEV_1_ from baseline in the EAR (PD_15_FEV_1_ allergen). In all subjects the PD_15_FEV_1_ allergen was re-challenged in single-step provocations on separate days to ensure that subjects met a 15% ± 5 drop in FEV_1_. If FEV_1_ was outside the expected range, the dose was adjusted, and the subjects were re-challenged until they met the objective of 15% ± 5.

After the screening visits the elected subjects started with the autovaccine treatment. Another four weeks after the intervention, the provocations were performed.

During the provocation period on the first visit (day 1) all subjects underwent methacholine challenge testing (MCT), induced sputum was collected, and a first, calculated, single-step dose of mite allergen (PD_15_FEV_1_) was inhaled. Next, on three consecutive days (visits 2–4), single-step challenges with PD_15_FEV_1_ allergen were repeated. On day 5 (visit 5) MCT and sputum collection were repeated. At each visit, pulmonary function and eNO were measured, a medication score was recorded, and peak-flow was measured after each visit hourly for at least ten hours to detect LAR. The LAR was defined as a 15% drop in PEF 3–7 hours post-challenge; subjects filled a protocol that was reviewed the next day.

All subjects were re-invited 18 months after visit 5 for a long-term follow-up.

### Pulmonary function test

Baseline pulmonary function tests were performed using the MasterScreen spirometer (CareFusion, Germany). The following parameters were recorded: forced vital capacity (FVC), forced expiratory volume in one second (FEV_1_), and Tiffeneau index (FEV_1_%FVC). For the FVC and FEV_1_ manoeuvres, ATS/European Respiratory Society test criteria for acceptability and repeatability [18] should be met in all measurements.

### Methacholine challenge testing

MCT was performed using the Aerosol Provocation System (APS, MedicAid-dosimeter, CareFusion, Germany). The APS dosimeter technique (CareFusion, Germany) allows computer-controlled production of aerosol using a jet-type nebuliser (Sidestream, MedicAid) powered by compressed air. The integrated pressure calibration procedure associated with the compressor ensures a highly constant and reproducible nebuliser output. The APS was calibrated to produce a continuous output of 240 mg/min. Several studies have shown particle size in terms of mass median aerodynamic diameter of approximately 3.2 μm, and an average of the fine particle fraction <5 μm of 49.7%.

MCT was performed, as described previously [19]. The doses of inhaled methacholine with a concentration of 16 mg/mL were increased according to the following pattern from step 1 to 5: 0.01, 0.1, 0.4, 0.8, and 1.6 mg. Thus, the entire protocol delivered cumulative doses of 0.01, 0.11, 0.51, 1.31, and 2.91 mg. Two minutes after each inhalation, spirometry was performed. The individual provocation dose (PD) causing a 20% drop in FEV_1_ (PD_20_FEV_1_) was calculated by logarithmic interpolation using an integrated programme.

### Specific allergen challenge

The Specific bronchial challenge with mite allergen was performed using the APS dosimeter technique (CareFusion Germany). Before each challenge, lyophilised mite allergen (Allergopharma KG, Reinbek, Germany) was resolved in 5 mL of 0.9% saline as a solution of 5000 SBU mL^-1^ (standardised biological unit).

### Incremental challenge

The incremental challenge protocol consistently followed the same algorithm, as described previously [2]. The dose of inhaled allergen was doubled beginning with the lowest dose of 10 SBU according to the following pattern from step 1 to step 5: 10, 20, 40, 80, and 160 SBU. Therefore, the entire protocol delivered cumulative doses of 10, 30, 70, 150, and 310 SBU. Ten minutes after each step-up, FEV_1_ was measured. Inhalation was stopped if FEV_1_ dropped ≥ 15% compared to the baseline values. The individual allergen dose that caused a 15% drop in FEV_1_ in the EAR (PD_15_FEV_1_ allergen) was calculated using a logarithmic interpolation between the doses before and after the 15% drop in FEV_1_ (Table 1).

**Table 1:** Subject’s characteristics

		**Total**
Subjects	[n]	27
Male/Female	[n]	12/15
Age	[yr, mean ± SD]	24.8 ± 4.0
FVC	[%pred, mean ± SD]	107.7 ± 10.4
FEV_1_	[%pred, mean ± SD]	103.4 ± 12.7
FEV_1_%FVC	[%, mean ± SD]	79.9 ± 8.2
Total IgE	[kU/L, mean ± SD]	165.9 ± 130.6
Spec. IgE D.pter.	[kU/L, mean ± SD]	26.3 ± 25.2
PD_15_FEV_1_ mite	[SBU, mean ± SD]	62.4 ± 53.6
PD_20_FEV_1_ metha	[mg, mean ± SD]	1.3 ± 1.1
eNO	[ppB, mean ± SD]	27.1 ± 14.6

### Single-step challenge

The single-step challenge was performed, as described previously [2]. Before each single-step challenge, the APS was programmed to deliver the individual dose of allergen that caused a 15% drop in FEV_1_ (PD_15_FEV_1_) by a technician or physician. At 10, 15, and 30 minutes after each challenge, spirometry was performed to determine the maximum decrease in FEV_1_ (EAR) compared to the initial values. A fall in FEV_1_ of 15% +/− 5 was accepted. If the drop was more than 20% or less than 10%, the single-step dose was adjusted according to the deviation of FEV_1_ and re-challenging doses were administered at least seven days apart. After the challenge procedure, patients received two to four puffs of Salbutamol (0.1 mg) until the FEV_1_ value returned to at least 90% of the baseline value. Peak flow was measured for up to ten hours following the challenge, and a LAR was defined as a drop of ≥15% PEF. Subjects received an action plan and rescue medication consisting of salbutamol and prednisolone.

### Measurement of exhaled NO

Measurements of exhalative NO were collected using the NIOX1 (Aerocrine, Solna, Sweden). NIOX1 measures eNO in exhaled air according to American Thoracic Society guidelines [20]. This chemiluminescence gas analyser is sensitive to eNO at concentrations ranging from 1.5–200 ppb and demonstrates a deviation from the mean value of +2.5 ppb at eNO <50 ppb or +5% of the measured value at >50 ppb.

### Sputum collection and processing

Subjects first inhaled 200 μg salbutamol and consecutively nebulised hypertonic saline at concentrations of 3%, 4% and 5% at intervals of every seven minutes. During this procedure, it was important to flush and clean the nose for disposal of the lower portion of squamous epithelium cells in the samples. Sputum was processed within one hour of collection. The selected sputum plugs were separated from saliva as much as possible, processed into a weighed Eppendorf tube, and processed with 4x weight/volume of 0.1% dithiothreitol (DTT). Afterward, 2 x weight/volume of phosphate-buffered saline (PBS) was added. Samples were filtered through 48-μm mesh and were centrifuged for 10 minutes at 790 x g. Cells were counted in a haematocytometer, and 60,000 cells per slide were prepared by cytocentrifugation (Cytospin 2; Shandon,. Runcorn, UK). To ensure the quality of the induced sputum one criteria is to keep levels of squamous epithelial cells low. Samples were considered satisfactory if there were less than 10% squamous epithelial cells. At least 400 inflammatory cells were counted for each specimen. Cells were expressed as percentages of the total cell count. The qRT-PCR cells were in RNAcell protect buffer (Qiagen (Valencia, CA).

### Quantitative Real-Time PCR (qRT-PCR)

Total RNA from sputum cells was extracted using the innuPrep RNA Mini Kit (Analytic Jena, Jena, Germany), according to the manufacturer’s instructions. In this assay, 5 μL of RNA was diluted 1:5 with RNase-free water, and the absorbance was measured to determine the amount of RNA. Before reverse transcription, a DNase treatment was performed using the DNase I kit (Qiagen, Hilden, Germany).

Reverse transcription of mRNA was performed in accordance with the manufacturer’s instructions (BioRad, Hercules, CA, USA). Briefly, probes were supplemented with 9 μL of a master mix of 1 μL iScript Reverse Transciptase (BioRad, Hercules, CA, USA), a random hexamer and oligo dT mix, 4 μL 10x iScript RT buffer and 4 μL nuclease-free water. These probes were incubated in a thermocycler at 25°C for an initial incubation step for 5 Min, followed by another step at 42°C for 30 Min and concluding at 85°C for 5 min.

Transcripts were quantified using two-step RT-PCR with Eppendorf Mastercycler Realplex S detection system (Eppendorf, Hamburg-Eppendorf, Germany) in Greiner 25 μL 96-well reaction plates (Greiner, Germany). The housekeeping gene glyceraldehyde-3-phosphate-dehydrogenase (GAPDH) was used as control in each plate. Primers for IL-5, IL-8, IFN-γ, and FoxP3 were designed by Qiagen (specific QuantiTect Primer Assays). PCR reactions were performed according to the manufacturer’s instructions in a final volume of 25 μl using QuantiTect SYBR Green Master Mix (Qiagen). Fluorescent product was detected at the last step of each cycle. A melting curve analysis was carried out immediately after amplification in accordance with the manufacturer’s instructions. Data expression and statistical analysis of genes involved in immune cells and inflammation markers were analysed.

Quantitative analysis was achieved based on the threshold CT value for each well and calculated using the Realplex S Database tool. The amount of mRNA expression was normalised with endogenous control GAPDH. The relative quantification and calculation of range of confidence were performed using the comparative threshold cycle 2^-ΔΔCt^ method (relative gene expression), as described by Livak and Schmittgen [21].

We have chosen to measure mRNA rather than protein levels because of the higher sensitivity of the qRT-PCR method compared protein measurements. In addition, there is no dilution effect using cells instead of supernatants after DTT-treatment of the sputum.

### Laboratory measurements

Serum was analyzed for specific antibodies against total IgE, specific IgE against against Dermatophagoides pteronyssinus IgE (D. pter), and sputum supernatants were measured for eosinophilic cationic protein (ECP) using a two-sided chemiluminescent assay (Immulite DPC, Bad Nauheim, Germany). Analysis was performed according to the manufacturer’s instructions. A total IgE of < 100 kU/L was estimated as normal and a specific IgE of > 0.70 kU/L was estimated as positive.

### Statistical analysis

For statistical analysis, GraphPad Prism 5.01 (GraphPad Software, Inc.) and Microsoft Excel were used. Pulmonary function parameters, eNO, PD_20_FEV_1_ methacholine, and cytokine expressions before and after the provocation period were expressed as arithmetic means and standard deviations (SDs). Differences between visits were analysed using Student’s paired *t*-test or Wilcoxon signed rank test, depending on normality assumptions and the homogeneity of variances. In the case of more than 2 visits, a non-parametric Friedmann test was used. A p-value of ≤ 0.05 was considered to be significant.

## Results

### Screening visits

Twenty-seven subjects, aged 18 to 40 years, were included. In advance, participants were screened for total IgE and specific IgE against house dust mite (D. pter.). Both mean values were elevated and the RAST class was at least II. All subjects underwent the incremental challenge with mite allergen. Baseline FVC values were 107.7% of predicted ± 10.4, and baseline FEV_1_ values were 103.4% of predicted ± 12.7 (Table 1). Mean PD_15_FEV_1_ dose in the incremental challenge was 62.4 SBU ± 53.6 (standardised biological units). The calculated PD_15_FEV_1_ was re-challenged in the first single-step challenge, and as expected [2], 18 of 27 subjects had a drop of maximum 15% ± 5 in FEV_1_. After adjustment of the dose, eight of the remaining nine subjects had a fall of 15% ± 5 in FEV_1_ with a second single-step challenge. One subject was re-adjusted and successfully challenged a third time. The mean drop in FEV_1_ in the successful challenges was 15.6% ± 4.3.

### Provocation period

After the screening visits all 27 subjects were gradually randomised into the intervention. Then the subjects were included in the provocations seven to 14 months (median eight months) after the screening visit. Of the subjects, seven did not fulfil the protocol; in single-step challenges, two subjects had a drop of < 10% FEV_1_; three subjects had a drop of > 50% FEV_1_ at visit 2; and in two subjects the baseline FEV_1_ was below 75% at visit 3. Two subjects had to stop the protocol, due to asthma attacks and the need of prednisolone during the night after visits 2 and 3, respectively. At least 18 subjects were included per protocol.

### Use of salbutamol and LAR

**Figure 2:**
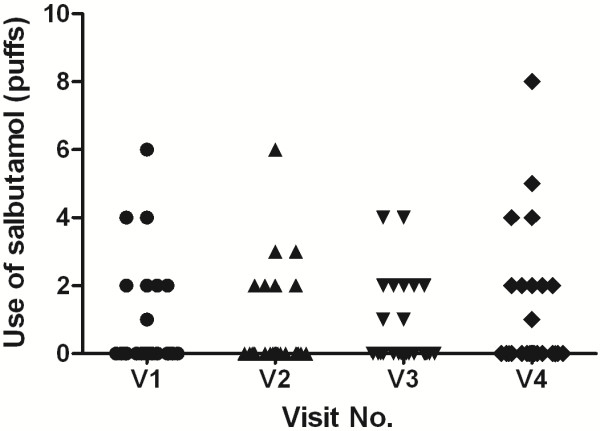
**Salbutamol use during the provocation period (overall mean 3.5 puffs).** The mean day to day use did not change significantly (p = 0.59). The bronchodilator use directly after the challenges is not included.

In the week before the provocation period, all subjects had no need for salbutamol, and during the intervention period (median eight months), only four subjects used salbutamol for asthma symptoms. During the provocation period, the subjects received a mean of 3.5 puffs salbutamol (SD 5.4) (Figure 2). Fifteen of 27 subjects developed a LAR after single-step challenges (Table 2). With the exception of four patients, all participants showed at least one day in pre-challenge baseline FEV_1_ a drop ≥200 mL compared with the initial value at visit 1.

**Table 2:** **LAR, use of Salbutamol, FEV**_**1**_**and eNO during provocation period**

		**V1**	**V2**	**V3**	**V4**	**V5**	**p**
LAR (all subjects, n = 27)	[n]	8	9	7	11		
Use of salbutamol (n = 27)	[puffs, mean ± SD]	0.85 ± 1.6	0.74 ± 1.5	0.74 ± 1.2	1.18 ± 2.0		0.59
Pre-challenge FEV_1_ (n = 18)	[absolute L, mean ± SD]	4.03 ± 0.93	3.86 ± 0.88	3.79 ± 0.99	3.83 ± 0.92	3.83 ± 0.90	<0.001
Pre-challenge FEV_1_ (n = 18)	[% pred, mean ± SD]	101.9 ± 15.6	97.4 ± 13.3	95.5 ± 17.6	97.1 ± 16.1	96.7 ± 14.7	<0.001
eNO (n = 18)	[ppb, mean ± SD]	29.4 ± 17.2	65.4 ± 31.2	93.5 ± 45.3	110.5 ± 51.4	114.5 ± 53.9	<0.0001

### Changes in PFT, BHR and eNO

**Figure 3:**
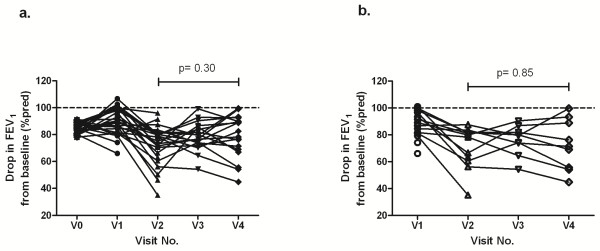
**Maximal drop in FEV**_**1**_**during the early asthmatic response after the single-step challenges.** (**a**). All subjects (n = 27), V0 represents the screening values. The mean drop in FEV_1_: V0, 15.6% ± 4.3; V1, 9.6% ± 9.9; V2, 17.5% ± 14.6; V3, 11.2% ± 10.5; V411, 2% ± 15.9. The values were not different between visits 2 and 4 (p = 0.30). (**b**). The 15 subjects that developed LARs. The mean drop in FEV_1_: V1, 11.8% ± 10.4; V2, 28.7% ± 15.3; V3, 23.8% ± 11.2; V4, 27.4% ± 18.9. The values were not different between visits 2 and 4 (p = 0.85).

In all subjects the mean drop in FEV_1_ during the EAR was 9.6% ± 9.9 at V1, 17.5% ± 14.6 at V2, 11.2% ± 10.5 at V3, and 11.2% ± 15.9 at V4. The drop during the EAR was not different between visits 2 to 4 (p = 0.30) (Figure 3a). Figure 3b indicates the 15 subjects that developed LARs. The mean drop in FEV_1_ during the EAR was 11.8% ± 10.4 at V1, 28.7% ± 15.3 at V2, 23.8% ± 11.2 at V3, and 27.4% ± 18.9 at V4 (V2-V4, p = 0.85).

**Figure 4:**
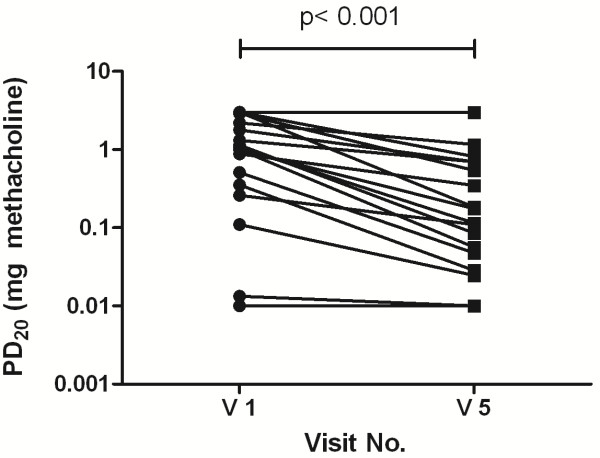
**The PD**_**20**_**FEV**_**1**_**methacholine on a logarithmic scale before and after the bronchial allergen challenges.** The PD_20_FEV_1_ decreased significantly from1.30 mg ± 1.08 to 0.45 mg ± 0.72 (p < 0.001).

**Figure 5:**
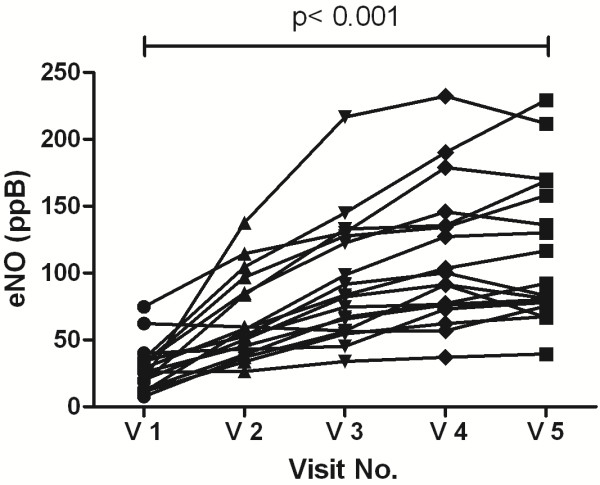
**Increase of exhaled NO 24 hours after the single-step challenges.** After each visit, the values increased stepwise. The data points are connected by lines that indicate individual courses of high, medium, and low responders.

In the 18 subjects who fulfilled the protocol the baseline FEV_1_ dropped from 101.9% ± 15.6 at visit 1 to 96.7% ± 14.7 at visit 5 (p < 0.001) (Table 2). BHR and eNO changes were highly significant. In the MCT, the PD_20_FEV_1_ methacholine dropped from 1.30 mg ± 1.08 at visit 1 to 0.45 mg ± 0.72 at visit 5 (p < 0.001) (Figure 4). The eNO increased consistently after every challenge from 29.4 ppb ± 17.2 to 114.5 ppb ± 53.9 (p < 0.001) (Figure 5, Table 2).

### Total cell count, eosinophils, ECP, and neutrophils in induced sputum

**Figure 6:**
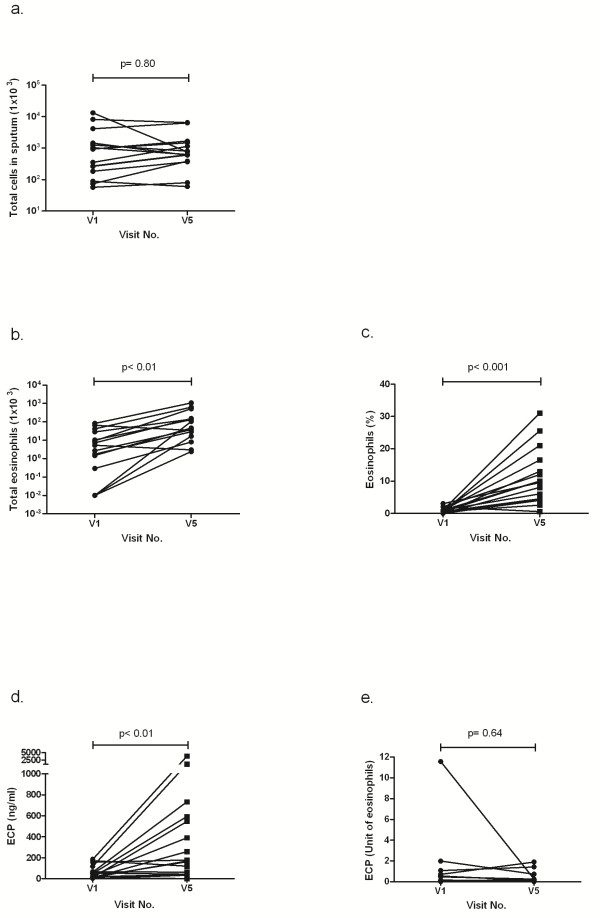
**Sputum cells and ECP before and after the bronchial allergen challenges.** (**a**). The total cell number was equally distributed in both samples (p = 0.80). (**b** and **c**). The total count and percentage of eosinophils increased significantly (p < 0.001). (**d** and **e**). The ECP increased significantly (p < 0.01), whereas the cytokine levels were unchanged in relation to the eosinophil cell counts (p = 0.64).

Induced sputum in 17 of 18 subjects was obtained and analysed at the beginning (V1) and at the end of the provocation period (V5). The numbers of the total cell count were equally distributed in samples before and after bronchial allergen challenges (median 927^.^10^3^ and 628^.^10^3^, p = 0.80) (Figure 6a). The total eosinophil count significantly increased from median 5.28^.^10^3^ to 46.2^.^10^3^ (p < 0.01) and the percentage of eosinophils from mean 0.9% ± 0.8 to 11.2% ± 8.5, respectively (p < 0.001) (Figure 6b,c). The levels of eosinophil cationic protein (ECP) increased from median 61.2 ng/ml to 523.3 ng/ml (p < 0.01) (Figure 6d), whereas the ECP expressed as per unit of eosinophils was unchanged (median 0.52 and 0.20 units, p = 0.64) (Figure 6e). The percentage of neutrophils did not change (9.8% ± 6.1 and 7.1% ± 6.0, p = 0.15) (data not shown).

**Figure 7:**
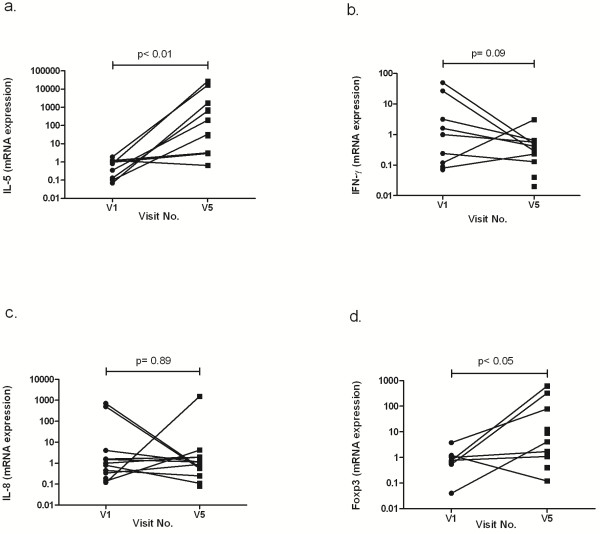
**Cytokine expression in the sputum before and after bronchial allergen challenges.** The values are related to the total cell numbers. (**a**). A significant increase in IL-5 expression (p < 0.01). (**b**). The IFN-γ expression did not significantly decline (p = 0.09). (**c**). The IL-8 expression was unchanged (p = 0.89). (**d**). A significant increase in Foxp3 expression (p < 0.05).

### Cytokine and Forkhead box protein 3 (Foxp3) expressions in sputum

The qRT-PCR showed a significant increase in IL-5 mRNA expression from 0.78 ± 0.19 to 4281 ± 2730 (p < 0.01), and a decreasing trend was observed in the expression of IFN-γ (from 7.78 ± 4.87 to 0.59 ± 0.28, p = 0.09). No differences were found in IL-8 mRNA expression (from 101.9 ± 240.0 to 131.3 ± 450.8, p = 0.89). In addition, transcription factor FoxP3 significantly increased after an allergen challenge from 1.05 ± 0.31 to 105.3 ± 65.37, (p < 0.05) (Figure 7a-d).

### Long-term follow up

All subjects were asked to return at a median of 18 months after the previous visit for a long-term follow up. As shown in Table 3, 22 of 27 subjects participated. None of the participants showed a deterioration of the lung function or changes in the total and specific IgE or the BHR.

**Table 3:** Long-term follow-up

		**V0**	**18 months**	**p**
Subjects	n = 22			
Male/Female	9/13			
FVC	[%pred, mean ± SD]	107.7 ± 10.4	108.5 ± 10.6	0.60
FEV_1_	[%pred, mean ± SD]	103.4 ± 13.2	100.5 ± 11.9	0.19
FEV_1_%FVC	[%, mean ± SD]	81.9 ± 6.5	79.2 ± 8.7	0.07
Total IgE	[kU/L, mean ± SD]	152.9 ± 107.9	166.2 ± 133.6	0.33
Spec. IgE D.pter.	[kU/L, mean ± SD]	28.7 ± 27.1	26.8 ± 22.6	0.90
PD_20_FEV_1_ metha	[mg, mean ± SD]	1.3 ± 1.0	1.0 ± 1.1	0.38
eNO	[ppB, mean ± SD]	26.1 ± 14.0	31.8 ± 21.4	0.25

## Discussion

Specific bronchial allergen provocation is an established tool in asthma research for proof-of-concept studies of new anti-inflammatory agents [16]. Repeated low-dose provocations imitate the natural allergen exposure. The standard high-dose allergen-inhalation challenge is usually performed to provoke the EAR and the LAR, and a proportion of asthmatics experience airway inflammation characterised by the presence of activated eosinophils [1]. Both standard and low-dose allergen provocations are safe when performed by experienced investigators and do not lead to persistent worsening of asthma or changes in airway function [16].

We aimed to induce airway inflammation and asthma symptoms by repeated high-dose provocation to establish a model that demonstrates the potency of a new agent to reduce severe asthma symptoms in small sample sizes. It is a matter of debate whether clinical studies of spontaneous asthma exacerbations should continue to be the gold standard for that end-point or if an agent might be proven in asthma volunteers and in small sample sizes under clinical conditions. Nevertheless, there are concerns about the feasibility and safety of this approach.

Moreover, it cannot be taken for granted that the worsening induced by four consecutive days of allergen challenge is relevant to the mechanisms in naturally occurring exacerbations. However, for asthma patients with severe allergies, it is impossible under certain conditions to escape repeated allergen exposures (e.g., Cladosporium allergy). These are patients who develop severe exacerbations despite therapy with high doses of inhaled corticosteroids and long-acting beta-agonists.

This study is the first to investigate the feasibility of this approach and the immune response after repeated high-dose bronchial allergen challenges with household dust mites in stable asthmatics within one week. We revealed a significant decrease of pulmonary function, an increased use of rescue medication and the development of LAR and BHR, as well as a significant induction of eosinophils and Th-2 cytokines.

It is important to determine whether the current protocol, which clearly results in at least a short-term progressive worsening of asthma, has a long-term impact on the future health of the individual. We re-investigated 22 of 27 subjects 18 months after the last visit. None of the participants showed deterioration of the lung function and, as shown by the specific IgE, no increase of the sensitivity to dust mites was noted. The BHR and the eNO, which were used as predictors of asthma severity and bronchial inflammation, were unchanged.

In the screening period, the subjects were re-challenged with the calculated PD_15_FEV_1_ in single-step challenges. In the first single-step challenge, 18 of 27 subjects exhibited a decrease in the FEV_1_ of a maximum of 15% ± 5. A correlation between incremental and single-step challenges has been confirmed in the literature [22,23]. The agreement of the decrease in FEV_1_ during the EAR revealed an intra-class correlation of 0.55. However, despite the good correlation, the authors describe relatively wide between-subject variabilities [22]. In the other study, 73% of the patients who demonstrated a 20% drop in the FEV_1_ during the incremental challenge produced a similar drop in the FEV_1_ in the EAR during a single-dose inhalation. Among the entire group, the repeatability according to Bland and Altman was poor (95% limits of agreement of −29.5 - 22.5%) [23]. The variability of the maximal decrease in FEV_1_ during the EAR, which is better expressed by the 95% limit of agreement than by correlation coefficients [24], was also shown in our previous [2] and present study.

Different bronchial provocation models show different outcomes regarding asthma symptoms and medication use. In repeated low-dose allergen provocations, night time asthma symptoms and night time β2-agonist use significantly increased during the challenging period, and the PC_20_ methacholine levels were significantly reduced [8]. In contrast, in another study using birch and grass pollen provocations, no patient experienced any significant early- or late-phase reactions [11]. Likewise, after inhalations of low doses of cat allergen, no patient experienced any asthmatic symptoms [25]. In a placebo controlled study with inhaled steroids, in 26 patients with mild asthma and mite allergy, repeated inhalations of the PD_5_ allergen were performed for two consecutive weeks. The β2-agonist use was significantly elevated in the placebo group, whereas there were no significant differences in the total daily symptom scores or in the FEV_1_ within or between the groups. In the placebo group, the PC_20_ methacholine levels did not decrease significantly after 2 weeks of allergen exposure [12]. In a similar protocol using the APS system, our working group showed that not all participants needed β2-agonists during the challenges with house dust mites despite of induction of allergic airway inflammation. The PD_20_ methacholine levels decreased in the placebo group, but the difference failed to reach significance [14]. In summary, in low-dose allergen challenges, subjects usually do not develop asthma symptoms and the elevated use of β2-agonists and day and night time symptoms are rare.

In repeated high-dose provocations, Rosenthal et al. [26] challenged patients who were allergic to ragweed on four successive days with stepwise inhalations of the antigen. A cumulative dose required for a 35% reduction in the specific airway conductance was calculated (PD35). In this study, only two patients had delayed, self-limiting reactions. Grainge and Howarth [15] established a model for repeated high-dose allergen challenges. They performed three provocations with the PD_15_ mite allergen at 48-hour intervals. The mean daily symptom score and the use of medication to relieve the symptoms during the week of the challenges increased significantly. With this design, the investigators did not observe serious adverse events, such as significant worsening of asthma symptoms requiring hospital admission, nor was there any requirement for the introduction of inhaled or oral steroids. Our model displayed a more aggressive approach. Seven subjects developed progressively more severe reactions, including two asthma exacerbations requiring oral steroid treatment. Four consecutive provocations with the PD_15_ dose at a daily interval led to an “overprovocation”, and such a protocol is not suitable for routine use or in experimental asthma research. However, high-dose provocations, e.g., the protocol suggested by Grainge and Howarth, are models that have been used in proof-of-concept studies to investigate the effect of asthma and antiallergic agents on symptoms and medication use.

In keeping with Grainge and Howarth [15], we found a significantly increased use of salbutamol and the development of LAR. The evaluation of the LAR using peak-flow meters might be disadvantageous because small changes in airway obstruction might be missed. However, two previous studies [27,28] showed that peak-flow measurements are a valuable tool to detect an LAR. The cut-off of a 15% decrease in PEF represents a significant LAR reaction, but values of less than 15% might be less sensitive than FEV_1_ monitoring. Furthermore, salbutamol was given after the allergen challenges. Short-acting ß-agonists reverse the LAR when it is not too severe; however, they do not prevent the LAR or any of the associated inflammatory events [16]. The protocol of this study is not suitable to reveal mild LAR, and the magnitude of the LAR might be biased. However, severe LARs are detected by PEF measurements and not prevented by ß-agonists.

Interestingly, with the exception of four subjects, all of the participants had at least one day with a pre-challenge baseline FEV_1_ ≤ 200 ml compared with the initial value, and the overall baseline FEV_1_ dropped significantly. Thus, the changes of PFT, the medication use, and the increase of BHR clearly demonstrate that the high-dose provocation model is an excellent tool to induce asthma worsening in stable step 1 asthmatics.

The finding that allergen exposure increased airway hyperresponsiveness to histamine or methacholine was first described in the classic publication by Cockcroft et al. [29]. A numerous number of subsequent publications confirmed the link between allergen-induced bronchial inflammation and BHR. Cartier et al. [30] were the first to demonstrate that the magnitude and duration of the decrease in PC_20_ histamine correlated with the magnitude of the LAR. As expected, we found a marked decrease in PD_20_ methacholine after the provocation period.

Kharitonov et al. [31] first reported that allergen-induced inflammation by single-allergen challenges caused an elevation in the level of eNO. In addition, during repeated low-dose allergen challenges, significant increases in eNO occurred [12,14]. We recently investigated the increase of eNO levels 24 hours after single-step challenges. Two challenges with the same amount of allergen were performed without worsening of asthma symptoms [2]. The intra-class correlation between both measurements showed substantial agreement (0.62, p < 0.001), whereas the limits of agreement revealed intra-individual variations.

In the present study, we demonstrated that repeated high-dose allergen challenges caused significant stepwise increases in eNO, beginning with the first provocation. It is somewhat surprising that an identical allergen dose, at least on the group level, reproducibly provided identical 15% drops in the FEV_1_, suggesting the development of tolerance. The development of tolerance implies an effect on Th-1 cytokines or a suppression of Th-2 cytokines. Thunberg et al. [32] investigated birch pollen-allergic patients and healthy controls and evaluated the local and systemic regulatory mechanisms in the EAR to bronchial allergen provocation. CD25 + CD4+ Treg cells were not as effective in their suppression of Th-2 responses, providing an explanation for the inability of infiltrating Treg cells to suppress Th-2 driven asthmatic responses to allergen provocation in the lung. By contrast, in inhaled and segmental bronchial challenges, IFN-γ levels did not differ between the asthmatic patients and the controls, and they were not affected 24 hours after allergen provocation [32,33]. However, Th-1-associated chemokines were present at 48 hours and may recruit a subsequent wave of Th-1-type lymphocytes that downregulate the on-going Th-2-type response [34]. In contrast, we found a decreasing trend in IFN-γ expression in repeated high-dose allergen challenges. Three repeated high-dose allergen provocations at 48-h intervals produced a similar decrease in the EAR and FEV_1_ in all challenges (p = 0.83), and there was no significant change in the baseline pre-challenge FEV_1_ (p = 0.53) [15]. It seems that, despite the induction of allergic inflammation, GINA I° asthmatics recover rather rapidly after bronchial challenges, as suggested by the constant pre-challenge lung function and the similar course of the EAR.

The induction of eosinophils by the allergen challenges is an important mechanism for the understanding of the allergic airway inflammation pathway and the link to LAR. De Monchy et al. [35] performed bronchoalveolar lavage (BAL) after allergen challenges in patients with asthma and in control subjects. In the BAL fluid of the patients, a significant eosinophilia was found, and the authors suggested that eosinophils and their mediators might be involved in the development of LAR after allergen inhalation. In two consecutive challenges with the same dose of allergen, the intra-class correlation coefficient for sputum eosinophils was 0.60 at 7 hours and 0.53 at 24 hours after the challenge [1]. The authors concluded that allergen inhalation challenges with measurements of sputum eosinophils are a noninvasive and reliable method to evaluate the anti-inflammatory effects of asthma therapies. In low-dose allergen challenges, a significant increase in the number of sputum eosinophils and/or ECP level was found [8,12,14,25]. The induction of eosinophils and ECP was more likely to occur in allergen challenges that provoked asthma symptoms or an increased use of salbutamol. However, it is surprising that the percentage of change in the eosinophil level in sputum at visit 5 was lower than that published by other groups 24 hours after a single high-dose allergen challenge [1,36]. Our results compare with similar changes in sputum eosinophils in low-dose allergen challenges [12,14]. In contrast, Sulakvelidze et al. [8] reported an increase in eosinophils of up to 21.2% in low-dose challenges. In high-dose allergen challenges, a percentage increase in eosinophils from 3.8% to 18.2% and from 7.2% to 31.8% was found, respectively [1,36]. In the former studies, the participants were mild asthmatics [12,14], whereas in the latter ones, the participants were screened to have an EAR and LAR [1,8] or 9 of 12 subjects had a definite late response [36]. The precondition of an LAR might have influenced the response after the allergen challenges, resulting in higher eosinophil levels.

We demonstrated that high-dose allergen provocation causes a significant elevation of eosinophils as well as a significant increase in the ECP levels. Therefore, proinflammatory effects might depend on the concentration and/or dosage of the inhaled allergen.

IL-5 is produced by Th-2 cells and is a key mediator of eosinophil activation. In low-dose allergen challenges, a significant increase in IL-5 was detected at day 5 [8], and a significant increase of the IL-5/IFN-γ ratio was only detected at day 19 [12], suggesting an increase in IL-5 and/or a drop in IFN-γ. In segmental allergen challenges in asthmatics, the IL-5 levels in BAL were low at baseline and increased after the challenge [33,34]. We demonstrated a significant increase in the IL-5 levels and an almost significant decrease in the IFN-γ levels after the provocation period. These results indicate that the allergen challenges induced IL-5 in either sputum or BAL.

The effect of the bronchial challenges on the balance of Th-1 and Th-2 is supported by our findings of Foxp3 upregulation. The appearance of Foxp3 suggests the involvement of CD25 + CD4+ Treg cells. Foxp3 is predominantly expressed in the CD25 + CD4+ Treg population, and Foxp3 expression in naïve T cells can convert these cells to a regulatory T cell phenotype that is functionally similar to naturally occurring CD25 + CD4+ Treg cells [37].

Most studies on immune regulation are performed on cells obtained from peripheral blood, which may not reflect the situation in the target organ. Thus, studies on the target organ of allergic asthma, i.e., the lungs, are essential [32]. Hartl et al. [38] reported that CD4^+^CD25^hi^ T cells were decreased in the BAL of asthmatic children compared with values in children with cough or control subjects, and the BAL CD4^+^CD25^hi^ T-cells from asthmatic subjects failed to suppress pulmonary Th-2 responses. The numbers of CD69+ and Foxp3+ lymphocytes were higher in the BAL after, compared with before, allergen provocation in asthmatic patients [32].

Foxp3 CD4^+^CD25^+^ Treg cells contribute to the control of allergen-specific immune responses in several major ways, e.g., the regulation of effector Th-1 and Th-2 cells [39]. Orihara et al. [40] showed that the Foxp3CD4 ratios correlated inversely at statistically significant levels with the serum total IgE level, eosinophil ratio, and serum IFN-γ level. Foxp3 as a marker for human Treg cells has some limitations, as T-effector cells transiently express Foxp3 after activation. However, CD4 + CD25 cells intracellularly stained for Foxp3 displayed significantly higher fluorescence intensity after, compared with before, allergen provocation. This finding indicates that the observed increase in infiltrating Foxp3+ cells corresponds to an increase in the number of functional Treg cells [32].

To the best of our knowledge, we are the first group to have shown that Foxp3 is expressed in sputum cells after bronchial allergen challenges, suggesting a modulating role of Treg cells after allergen exposure.

Repeated high-dose bronchial allergen challenges did not influence neutrophilic inflammation. In our study, as well as in the study by de Kluijver et al. [12], the number of neutrophils and the levels of IL-8 or neutrophil elastase in sputa were not affected by high- or low-dose allergen challenges, respectively.

## Conclusions

High-dose allergen challenges caused significant asthma worsening, increased salbutamol use, and induced BHR. Moreover, a marked Th-2 mediated inflammation involving eosinophils and high NO was demonstrated. The high-dose challenge model is suitable only in an attenuated form in disease volunteers in proof-of-concept studies and in clinical settings to reduce the risk of severe asthma exacerbations.

## Competing interests

The authors have no non-financial or financial interests to declare.

## Authors’ contributions

JS participated in the design of the study, he performed the statistical analysis and drafted the manuscript. SV carried out the bronchial provocations, the sputum collection and processing and helped in the statistical analysis. UZ performed the real-time PCR. MAR participated in the design of the study and interpretation of data. SZ conceived of the study, participated in its design and coordination and helped to draft the manuscript. RS participated in its design and coordination, performed the real-time PCR, helped in statistical analysis and to draft the manuscript. All authors read and approved the final manuscript.
